# Detection of Semi-Solid Materials Utilizing Triple-Rings CSRR Microwave Sensor

**DOI:** 10.3390/s23063058

**Published:** 2023-03-12

**Authors:** Ahmed Jamal Abdullah Al-Gburi, Norhanani Abd Rahman, Zahriladha Zakaria, Merih Palandoken

**Affiliations:** 1 Centre of Telecommunication Research & Innovation (CeTRI), Fakulti Kejuruteraan Elektronik dan Kejuruteraan Komputer, Universiti Teknikal Malaysia Melaka, Durian Tungal 76100, Malaysia; 2 Department of Electrical Engineering, Politeknik Port Dickson (PPD), Port Dickson 71250, Negeri Sembilan, Malaysia; 3 Department of Electrical and Electronics Engineering, Izmir Katip Celebi University, 35620 Izmir, Turkey

**Keywords:** semi-solid, complementary split ring resonator (CSRR), triple-rings CSRR, polypropylene (PP) tube, Q-factor, sample under tests (SUTs), sensitivity

## Abstract

This article proposes the design, fabrication and measurement of a triple-rings complementary split-ring resonator (CSRR) microwave sensor for semi-solid material detection. The triple-rings CSRR sensor was developed based on the CSRR configuration with curve-feed designed together, utilizing a high-frequency structure simulator (HFSS) microwave studio. The designed triple rings CSRR sensor resonates at 2.5 GHz, performs in transmission mode, and senses shift in frequency. Six cases of the sample under tests (SUTs) were simulated and measured. These SUTs are Air (without SUT), Java turmeric, Mango ginger, Black Turmeric, Turmeric, and Di-water, and detailed sensitivity analysis is conducted for the frequency resonant at 2.5 GHz. The semi-solid tested mechanism is undertaken using a polypropylene (PP) tube. The samples of dielectric material are filled into PP tube channels and loaded in the CSRR centre hole. The e-fields near the resonator will affect the interaction with the SUTs. The finalized CSRR triple-rings sensor was incorporated with defective ground structure (DGS) to deliver high-performance characteristics in microstrip circuits, leading to a high Q-factor magnitude. The suggested sensor has a Q-factor of 520 at 2.5 GHz with high sensitivity of about 4.806 and 4.773 for Di-water and Turmeric samples, respectively. The relationship between loss tangent, permittivity, and Q-factor at the resonant frequency has been compared and discussed. These given outcomes make the presented sensor ideal for detecting semi-solid materials.

## 1. Introduction

In the last few years, there has been an incendiary development of interest in microwave resonator sensors for different technological challenges, such as detecting and characterising the properties of solid and semi-solid materials with their configuration sensing analysis. Microwave sensors are among the numerous widely used sensors that have been operated for material characterization in farming, medicines, and industry [1,2,3]. Material characterization is essential when looking at the qualities of a material, whether it is a solid or a powdered sample [4,5]. The sensitivity of a microwave sensor can be operated to characterize material qualities. Compared to solid materials, the permittivity of fluid materials is inclined to be influenced by aspects such as temperature, humidity, impurities in the experimental specimen holder, atmospheric pressure, and others [6,7,8]. Furthermore, experiments with a fluid specimen are less suitable due to their liquid behavior. Numerous liquid specimens comprise polar particles, showing high dielectric constant and loss of attributes. On this point, the development of in situ experiments on dielectric constant and loss tangent for liquid materials constitutes a dynamic market [9].

A resonator or sensor is a device, module, or subsystem that detects occurrences or differences in its surroundings and transfers data to other electronics, most typically a computer processor. Over the last decade, precise material characterization measurement has become increasingly critical. Food quality control, bio-sensing, and subsurface detection have all profited from examining a material’s composition and properties, and their physical and chemical transformations [10,11,12]. Material characterization arrangement designs rely laboriously on resonant techniques, which can be divided into two categories: resonator and profound disturbance [13,14]. Compared to wideband methods, resonant techniques can represent a material’s characteristics proposed at an individual frequency or a discrete set of elevated precision frequencies. Microwaves, insulating materials, and coaxial sensors have commonly been employed to characterize materials in various topologies [15,16,17]. These techniques are constructed to fulfil the industry and market utilization due to their ability to be used for highly sensitive materials. Different dielectric characteristics of substrates can characterize the sensor, such as transmission and reflection coefficient features [18,19,20]. However, the adequate performance of microwave sensors is still not saturated and challenging in dielectric material characterizations. On the other hand, this sort of measurement is frequently too complex for industrial use. Planar resonator sensors are used in this situation, as they are used in contemporary uncomplicated permittivity measurements and are easy to use [21,22]. Following the planar sensors approach, material characterization was conducted utilizing precision sensitivity and high Q-factors, as reported in [23,24]. On the other hand, some resonator sensors are convoluted, pricey to build and demand many techniques to be detected [25,26,27,28]. These techniques result from low sensitivity and Q-factor matters, restricting the material’s characterization measurement.

Numerous configurations have been used to define the characteristics of the specimens to be experimented on and tested, such as complementary split ring resonators (CSRRs), which are considered the most typically utilized ones in the structure of liquid microwave sensors [29,30,31,32,33,34,35,36]. In [29], the sensor was designed and developed to maintain the liquidity of the fluid during experiments, leading to a large amount of loss of fluid liquid under tests (LUTs). Microwave sensors proposed by Kiani et al. [30] can effectively evaluate the dielectric constant of fluids while not the tangent loss of the liquid material. The sensor suggested by Su et al. [31] employs flexible fabrics which can only be used in experiments with low-loss materials. At the same time, the sensor’s sensitivity in [32] is too low, about 150 MHz/mgmL^−1^, and much noise can be noticed throughout the measurement process. A solid planar microwave sensor based on SRR is proposed in [33]. The proposed resonator had a Q-factor of 240 at 2.3 GHz with total dimensions of 50 × 40 × 0.79 mm. A low-profile microwave vector method suggested in [34] has the benefit of a single transmission line to enhance the sensitivity, which permits measuring the value and phase of the material under test. Another study was presented in [35] for material liquid detection. The sensor was designed based on the TG-CSIW technique and promised a very high Q-factor of 700 at 2.45 GHz. The TG-CSIW sensor size was 69 × 69 × 1.45 mm. Lastly, in [36], a novel GWCR approach was investigated for liquid detection. The stated sensor was tested and measured for various fluid concentrations, such as ethanol and methanol, with a sensor size of 38 × 35 mm, and the sensitivity was only 0.156.

This paper proposes a single-band microwave sensor integrating a CSRR configuration and DGS ground plane to structure the triple-rings CSRR sensor, which is operated at 2.5 GHz. The proposed sensor is employed for semi-solid material characterizations. The total dimensions of the modelled triple-rings CSRR resonator are only L × W × h of 25 mm × 20 mm × 1.52 mm. The modelled sensor offered a high sensitivity of about 4.806 with a high Q-factor of 520 at 2.5 GHz. Through careful investigation and measurements, the suggested sensor can recognize the SUTs topology and determine their concentrations.

## 2. Triple-Rings CSRR Design and Validation

### 2.1. Sensor Design Configuration

**Figure 1:**
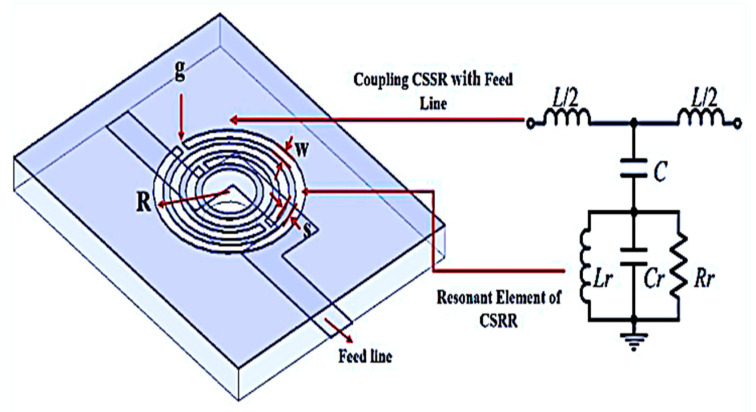
Triple-rings structure and its equivalent circuit.

The structure was designed based on the basic geometry of CSRR explained by [37], and the antenna design concept was suggested by [38]. It proves that the circular CSRR provides better sensitivity in comparison with the rectangular CSRR having the same unit area. The resonant circuits of the sensors should have a high Q-factor and small size in order to ensure high accuracy and sensitivity of the analysis. The Roger RT/Duroid 6002 substrate is chosen for its small dielectric loss factor of 0.0012 due to its weak material conductivity in strong dielectric fields. It is ideal for large-band applications where losses must be reduced. The designed transmission line width is 2.1 mm, with the substrate and copper cladding thickness of 1.52 mm and 0.07 mm, respectively, to improve the sensitivity of the sensor device, which can fit several types of SUTs due to its large-scale sensor region. The triple-rings CSRR of the resonance frequency is analyzed by a quasi-static and equivalent circuit model, as described in Figure 1. The gap and the shape of the ring perpendicular to the gap represent the inductance, while the ring generates a capacitance. Numerical simulation can be used to compare the sensitivity of the planar CSRRs based on each ring to study the losses in the resonators, as the fundamental factor for degrading the Q-factor of the resonators.

The slit gap is one of the main parameters for triple-rings CSRR. If the slit is removed, the ring will not generate a particular resonance frequency. The capacitance of the CSRR (*C_CSRR_)* structure etched at the ground plane is due to the metallic strip between the slots, and inductance (*L_CSRR_*) is due to the space between the metallic strips. The geometrical structure of SRR and CSRR is approximated by Equation (1). It can be determined for certain standard physical variables such as ring resonator diameter, effective dielectric constants, and feedlines length. A current that flows along the ring produces a magnetic field that travels through the ring, which functions as an inductance. Various gaps in the ring and the spacing between the rings serve as capacitance factors.

The resonance frequency, inductance, and capacitance values of the CSRR are determined following [39]:

(1) $$f=\frac{1}{2\pi \sqrt{L_{CSRR}C_{CSRR}}}=2.57\;\mathrm{GHz}.\;$$

where the value of *C_CSRR_* is 0.98 𝑝𝐹 and *L_CSRR_* is 3.88 𝑛𝐻.

**Figure 2:**
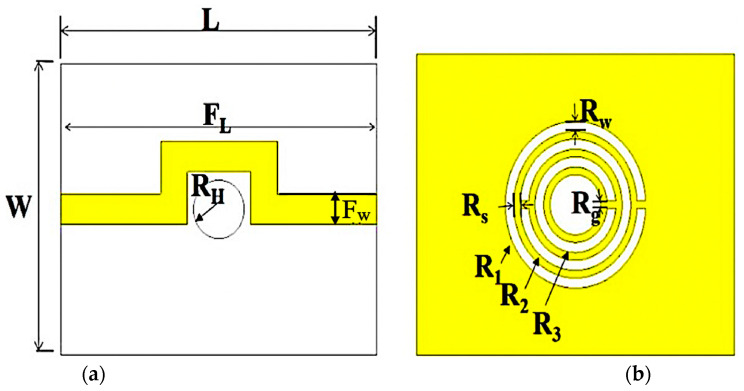
Triple-rings sensor design structure. (**a**) Top view of the transmission line position. (**b**) Bottom view defect ground structure of triple-rings CSRR.

The parameters of the outer radius of the ring triple-rings *CSRR* are the radius of the ring (*R*), which is 5.54 mm, the distance between slots (*S*) equal to 0.5 mm, and *W* = 0.68 mm as the slot width. A coupling gap of 0.5 mm is the main element determining the ring structure’s capacitance strength, while the current flow around the ring creates an electric and magnetic field due to the patch’s behavior. The hole between the curve-feed line (R_H_) and the CSRR structure carved on the ground will regulate the capacitance power. The divided ring excitation form determines the power of the inductance, which decreases when the number of divisions rises. To increase flux density, the design structure of the planar sensor is to be improved. The range dimension of the curve-feed sensor in Figure 2 is 25 mm × 20 mm × 1.52 mm (*L* × *W* × *h*).

Several SUTs were tested using the proposed curve-feed sensor. To avoid any undesirable failures during the measurement, room temperature must be consistent. Responding to the electromagnetic properties of the sample, resonant frequency, insertion loss, and Q-factor differ.

The design structure has many advantages over the traditional SRR, particularly for the analysis of the SUT’s properties. The design structure also theoretically increases the electrical field propagation strength in the sensing area. In the middle of the curve U-shape of the transmission line (top copper) and the triple-rings (lower copper–ground structure), the resonator sensor has been restructured to maximize the amount of electrical flux with the presence of SUTs. For this purpose, the sensor was developed with a high Q-factor in order to achieve sample sizes with a small quantity.

The current around the ring produces a magnetic field travelling via the ring. Only apparent magnetic coupling with limited radiation loss can be made by introducing multiple rings to the structure. The triple-rings structural design idea is to create interactive ring elements that are less than the electromagnetic radiation added. It raises the quantity of electric flux around the rings for the sensor. Table 1 describes the approximation method as well as the dimensional geometrical requirements for the Triple Rings sensor.

**Figure 3:**
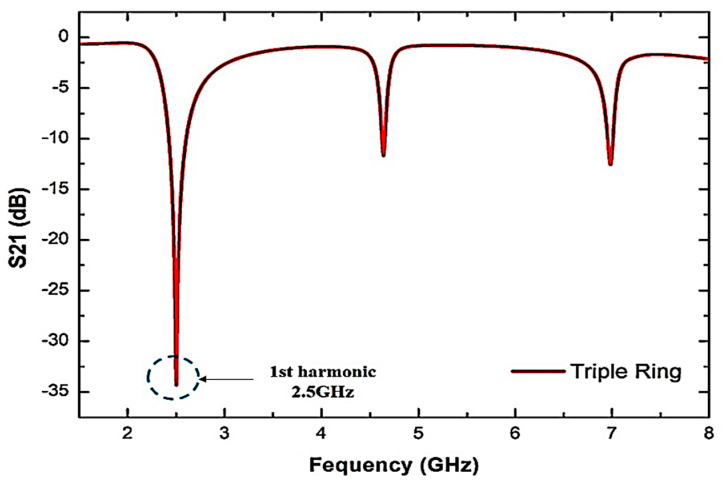
Triple-rings sensor simulated frequency response.

Figure 3 shows the simulation response of the triple-rings resonator design. The model response works in a comprehensive system of two-port networks supporting the analyzer’s input and output. The reaction will normalize the interests in order to obtain reasonable resonators and further avoid undesirable signal output and achieve acceptable frequency.

As can be seen from Figure 3, the maximum response of the resonant frequency (𝑓) at 2.5 GHz is the best performance. The Q-factor and insertion loss, S_21_, of the triple-rings sensor are 520 and −34.281 dB, respectively. The result of the adjustment of some sensor variables is to satisfy the purpose of design efficiency. In order to obtain a particular resonant frequency, parametric experiments have been carried out already when the TRs compact resonator has similar actions as the single and double-ring versions, and the procedure should be more straightforward.

Hence, it is possible to predict the physical parameters used for modifications designed to achieve a satisfactory response to the structure. The extra ring design is intended to test the effect of another split structure on the sensor’s response. The triple-rings are configured at 2.5 GHz with a very large Q-factor (>400) even when the inductance value has been reduced because of the increased split structure.

**Figure 4:**
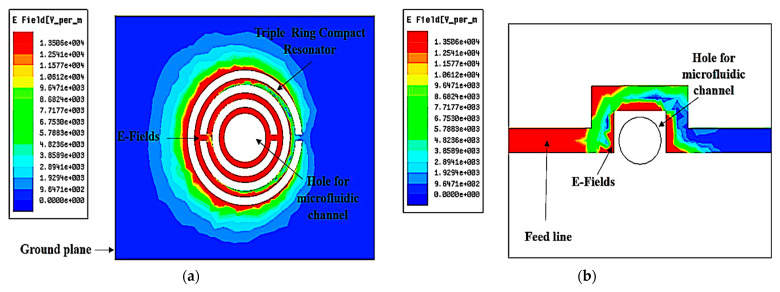
Distribution of triple-rings sensor electric fields. (**a**) Ground plane. (**b**) Feed line.

Figure 4 shows an E-field increase as an EM signal spreads through the sensor. The added split ring decreases the quality factor and raises the frequency bandwidth. The performance of the system is therefore reduced. The polar structure of the SUT will be influenced by maximum electrical flux density 1.5506E + 04 V/m, towards the sensing identification, providing an electrical reaction dependent on a variety of variables.

### 2.2. Parametric Study on Triple-Rings CSRR Microwave Sensor

The triple-rings CSRR sensor is designed using a CSRR etched at the ground plane, as illustrated in Figure 4a. A Curve-feed CSRR sensor is constructed and simulated to resonate at 2.5 GHz with a quality factor of 520. The defects on the ground plane or defect ground structure (DGS) interrupt the current distribution of the metallic plane; this interference affects the properties of a transmission line (or any structure) by adding specific parameters (slot resistance, slot capacitance, and slot inductance) to the line parameters (line resistance, line capacitance, and line inductance). Among specific terms, each fault engraved under the microstrip line in the ground improves the efficient capacitance and inductance of the microstrip line when applying slot resistance, capacitance, and inductance [39]. DGS is beneficial to the sensor design since this structure can reduce the overall size of a specific planar structure when providing optimum performance in microstrip circuits. Thus, this methodology helps miniaturize the overall dimension of the planar circuits. The disturbance will alter the characteristics of a transmission line, for instance, [40].

**Figure 5:**
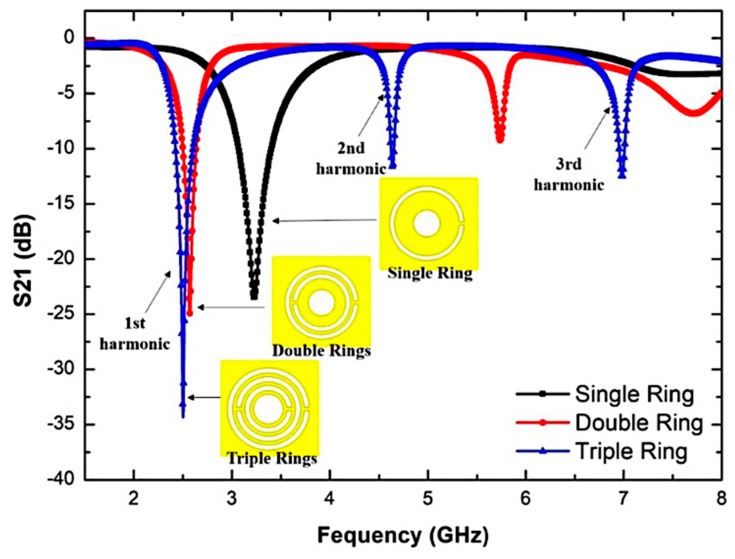
Simulated insertion loss characteristics of a single, double and triple-rings CSRR sensor.

The investigation on the triple-rings CSRR sensor is based on single rings, double rings, and triple rings. Figure 5 demonstrates the insertion loss characteristics of the number of triple-rings CSRR from a matching inset picture that describes the geometries of the sensor. The resonance frequency of a single ring is 3.23 GHz, while double and triple rings shifted to 2.57 GHz and 2.5 GHz, respectively. Hence it is noticed that with the increasing number of rings, the resonance frequency will be moved to a lower frequency, and more energy concentration will be offered via the electric field, thus increasing the sensitivity of the sensor. The parametric study also demonstrates that the slit effect between the ring on CSRRs provides a new resonance frequency. Therefore, it is able to improve the multiband.

The data in Table 2 reveal that the Q-factor and electric flow intensity were subsequently improved by the enhancement of the unit split structure. It indicates that the sensitivity increases because of the capacitance and the inductance strength. The flux density of single, double, and triple CSRR are increased from 9.8858E + 03 V/m to 1.3347E + 04 V/m, accordingly. Therefore, the selection of a triple ring for this design is very appropriate because it produces stronger e-fields for sensors. The triple-rings CSRR sensor has a high Q-factor, and it can test more than one type of SUT and build a strong electric field.

### 2.3. Analysing of the Sample under Tests (SUTs)

**Figure 6:**
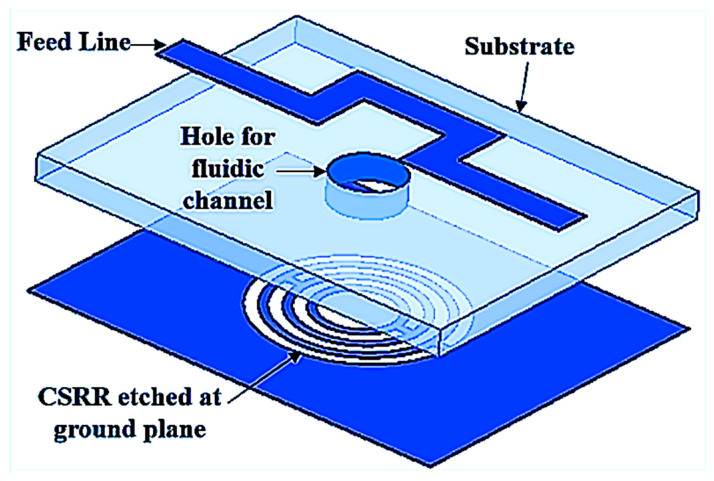
Perspective view of triple-rings CSRR sensor.

The Sample under tests (SUTs) is discussed and demonstrated in this subsection. The triple-rings CSRR sensor is designed and analyzed based on the CSRR structure etched at the ground plane with the curve U-shaped feed line for the strong electromagnetic excitation around the hole, as shown in Figure 6. In order to prove the concept of design, numerous simulation analyses were conducted by testing the SUT on sensor capabilities.

#### 2.3.1. Effect of Polypropylene (PP) Based Triple-Rings Sensor

**Figure 7:**
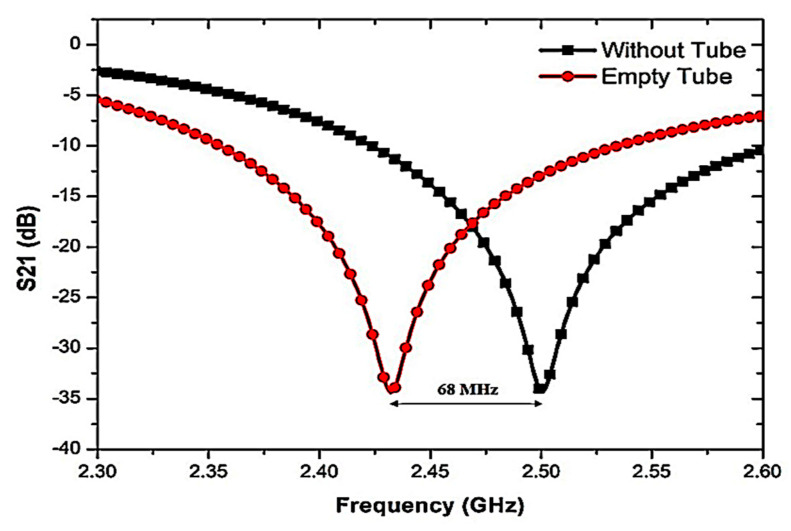
The effect of frequency shifted when the pp tube was loaded into the triple-rings CSRR sensor.

In order to prove the concept of design, numerous simulation analyses were conducted by testing the SUTs on sensor capabilities. The semi-solid testing mechanism is performed for SUT characterization using a Polypropylene (PP) tube. The samples of dielectric material are filled into PP tube channels and loaded in the CSRR center hole. The e-fields near the resonator will affect the interaction with the SUT. From the observation, it shows that when the empty tube is loaded, the resonant frequency is marginally changed to a lower frequency at 2.432 GHz with 68 MHz bandwidth, as indicated in Figure 7.

**Figure 8:**
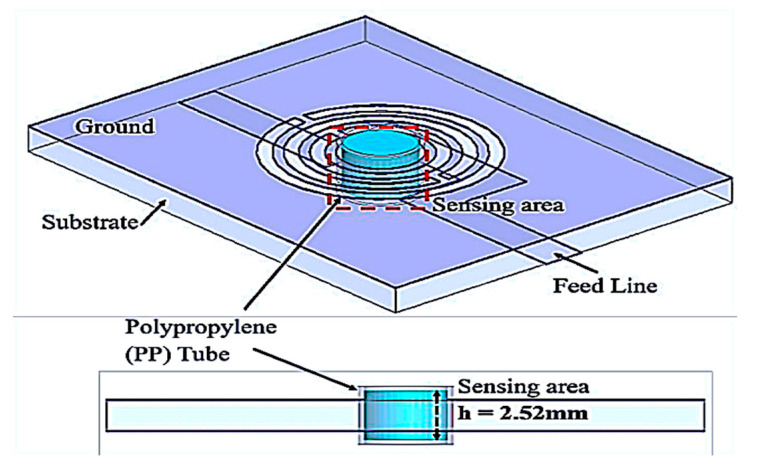
PP tube loaded into the substrate for 7.92 μL at a time.

To evaluate the sensing area of the tube, the sample volume uses characterization based on the sensor thickness and maximum electric flux located. The volume calculated follows Equation (2), and is illustrated in Figure 8, showing the close-up image to show the sensing region. The best performance can be produced by using the volume lengths of the tube when the average frequency change exceeds a single saturation level (*h*).

𝑉 = 𝜋𝑟^2^h (2)

where *r* is the radius of the fluidic channel and *h* is the height of the sensing area based on the saturation level of volume.

**Figure 9:**
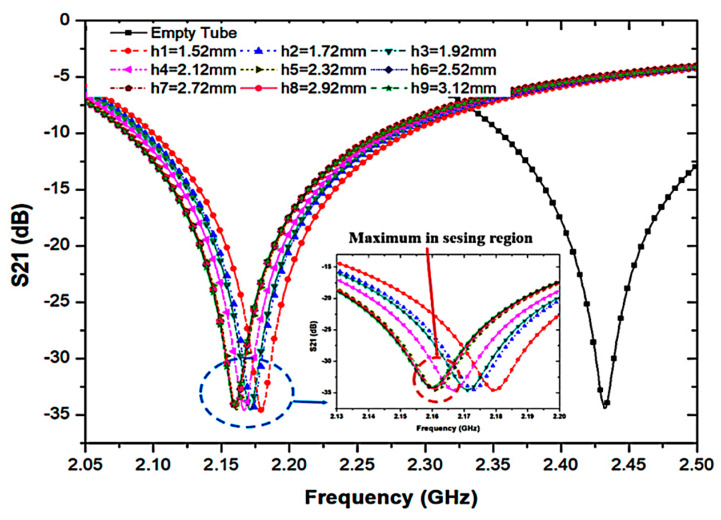
Simulated frequency shift at different volume levels of the sensing area using distilled water filled in the PP tube.

The simulated transmission coefficient (S_21_) of the proposed sensor with the empty and distilled water (DI-water) loaded into a 6 mm tube is indicated in Figure 9, where the optimal volume length is 2.52 mm, equivalent to 7.92 μL of semi-solid.

#### 2.3.2. Simulation of Semi-Solid Materials under Test

**Figure 10:**
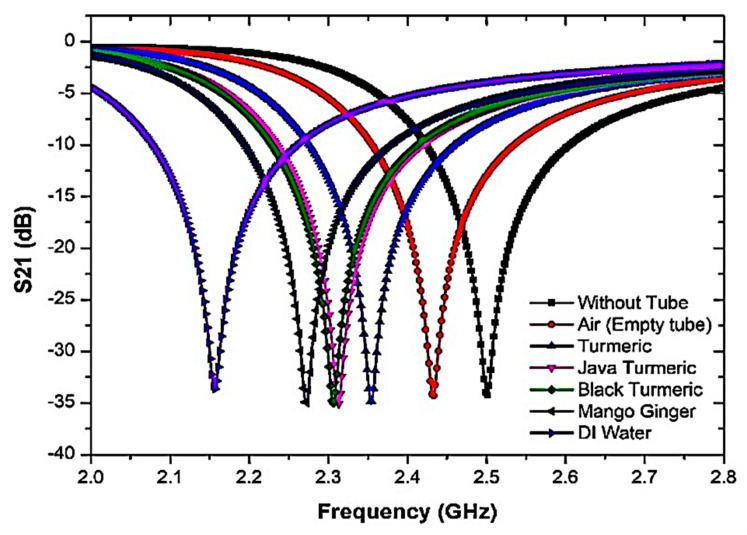
The frequency response of triple-rings CSRR sensor with the presence of SUT samples.

To further analyze the sensor response towards the triple-rings CSRR sensor, several semi-solid SUTs with various dielectric properties and relaxation periods have been used. These SUTs are Air (without SUT), Java turmeric, Mango ginger, Black Turmeric, Turmeric, and Di water. The resonant frequency was also measured with and without SUT. Every sample has dielectric properties that disturb electric fields within the sensing region and is ultimately described in response to the characterization of the properties. Figure 10 shows that due to the polar existence of samples, the resonant frequency and insertion loss were explicitly modified.

The constant temperature monitoring and numerous sample tests are standardized, and the average test values are measured accurately. In order to secure the same outcome that depends on the theoretical principle, a slight frequency difference is detected and critically compared with the measured data.

The analyses on both port networks perceived the importance of the interference response and transmitted information to identify dielectric properties. Furthermore, by using the permittivity value in Aziz et al. [41], the semi-solid samples of Java turmeric, Mango ginger, Black Turmeric and Turmeric are 𝜀′ of 34.52, 45.6, 46.68 and 58.61, respectively, at less than 2.5 GHz resonant frequency. In addition, concerning the dielectric properties of the present samples, the quality factor of the compact resonator-sensors was decreased. The high permittivity value leads to a lower change in frequency due to capacitance and inductance capacity, as illustrated in Figure 10. Consequently, the Q-factor of the samples differs according to the various dielectric properties. Table 3 shows the results of the frequency response analyses when SUTs are used.

## 3. Fabrication, Measurement and Characterizations

### 3.1. Curve-Feed Sensor Fabrication

**Figure 11:**
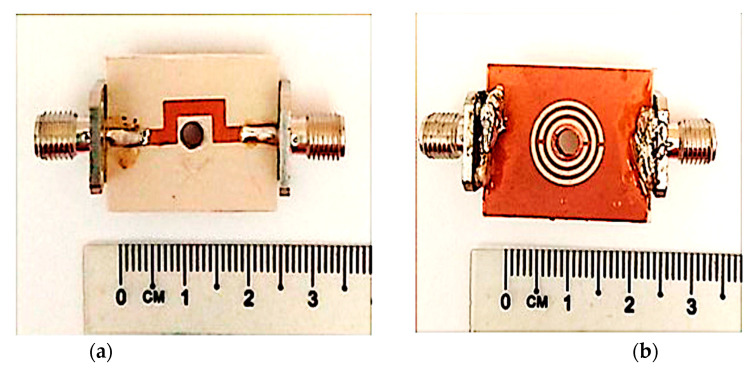
Fabricated prototype of triple-rings CSRR sensor. (**a**) Top and (**b**) bottom views.

As part of this research, the fabrication and sample preparation for measurement is prepared for the sensors’ validation in this work. This includes the fabrication of the triple-rings CSRR sensor using Roger RT/Duroid 6002 substrates with a geometrical width of 20 mm × 25 mm × 1.52 mm (𝑤 × 𝑙 × h) through the standard photolithography technique and PCB etching method. The image of the sensor produced is shown in Figure 11 and has a relative permittivity, 𝜀′ of 2.94 and loss tangent, *tan δ* of 0.0012. However, the finishing between connector type radial 50 Ω straight flange mount SMA and PCB board did not give good grounding which will contribute to a high tolerance. Therefore, it is recommended to use connector type RF solution 50 Ω straight edge mount SMA in the future to provide better grounding and give a minimal tolerance. 

**Figure 12:**
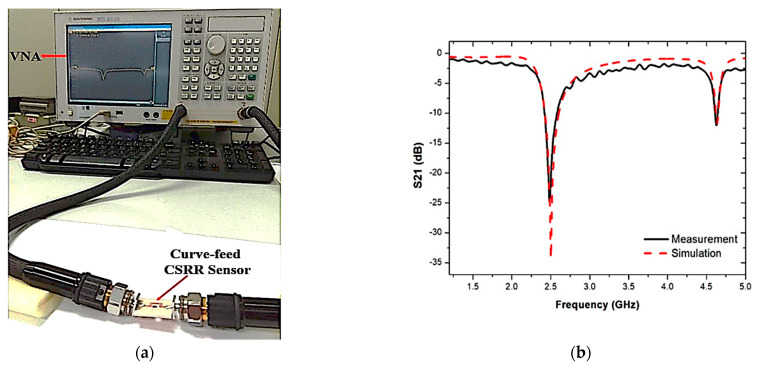
(**a**) Experimental setup. (**b**) Simulated and measured results of triple-rings CSRR sensor.

The perturbation parameters of the loaded transmission line are measured by employing Vector Network Analyzer (VNA). The sensor response is assessed and recorded during the experiment when filled with different SUTs. These SUTs have been mounted on the curve-feed CSRR sensor to evaluate the dielectric materials of solid samples. In contrast, the solid samples are placed over the CSRR structure of the ground sensor. The experimental setup of the triple-rings CSRR sensor with the S-parameter results for simulated and measured frequency responses is shown in Figure 12. The Q-factor of the proposed sensor was found to be 520 at 2.5 GHz, with −34.281 dB of insertion loss performance.

**Figure 13:**
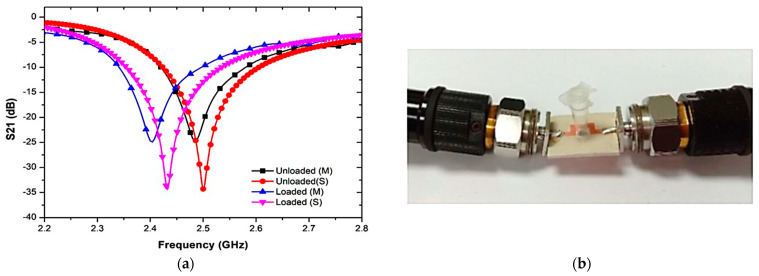
(**a**) Simulated and measured results of output load and unloaded tube as an empty sample (air), (**b**) PP tube loaded into triple-rings CSRR sensor.

Figure 13 shows the prototype of the proposed sensor and the S-parameter of the comparison between the simulated and measured responses when the PP tube was loaded into the triple-rings CSRR sensor. The graph showed some differences in both simulation and measurement results. Due to the fabrication errors giving a discrepancy between the simulated parameters and during the manufacturing process, this changes the frequency response. The findings of the measured results clearly show the resonance frequency, quality factor and insertion loss, S21, lower than the simulation as tabulated in Table 4. The weak connectivity of the port couples may lead to radiation loss within that input and output port network. Therefore, simulation and manufacturing enhancements will be investigated in order to minimize these errors.

Therefore, the resonance sharpness is calculated by the Q-factor. The higher the Q-factor, the narrower the resonance peak, so the sensor becomes more sensitive with the value of 520 for the unloaded sample.

Additionally, the semi-solid sample from Zingiberace families was brought from the market, namely Java turmeric, Mango ginger, Black turmeric, and Turmeric. The market is an ideal place to purchase the sample as it has many choices, and the sample has to be fresh. The sample was placed in a black plastic bag to avoid sunlight to maintain the freshness of the samples before starting the experiment. The samples were cleaned with tap water followed by distilled water to remove dust, then they were peeled to remove the skin and finely cut into small pieces (grinding) and inserted into the tube. The SUTs of the semi-solid samples were used before they were compressed in the 6 mm diameter PP tube with a minimum sense of volume length of 2.52 mm, which is equivalent to 7.92 μL.

Several semi-solid SUTs were measured to validate the sensor efficiency from 1 to 5 GHz using the Agilent Vector Network Analyzer. The solid sample was placed over the sensor, and the semi-solid was loaded into a PP tube. The tube contains a total amount of semi-solid of 7.92 μL filled by the sensing region. The sample handling is also easy, and repeated analysis can be carried out easily. In addition, the validity of the data results was checked by contrasting the measured data between the proposed sensor and the existing commercial sensor (Agilent 85070E dielectric probe kit). Three times repeated measurements produce the average data values at room temperature. The frequency response shift is evaluated and objectively compared with the simulated results to maintain the same performance. The polynomial fitting technique is used, and the numerical expression is created from these specific data sets. The working principle contributes to identifying the complex permittivity, loss tangent, concentration and sensitivity of the proposed sensor.

**Figure 14:**
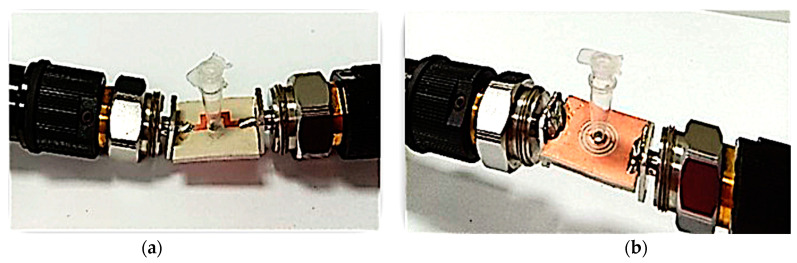
Position of PP tube loaded at the center of Curve-feed CSRR sensor, (**a**) Top and (**b**) bottom.

**Figure 15:**
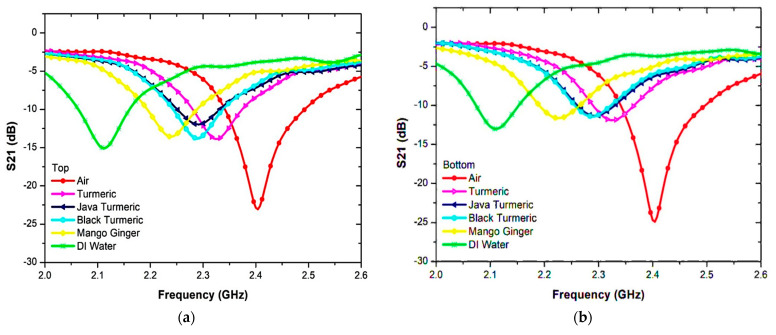
Measurement results of Semi-solid SUT channel location (PP tube). (**a**) Top sensor. (**b**) Bottom sensor.

The PP tube position analysis for SUT filling was identified before the measurement and analysis of the permittivity. It can be measured at any position, either the top or bottom of the Curve-feed CSRR sensor, as described in Figure 14. The PP tube analysis was conducted on the semi-solid samples and the resonant frequency readings were similar. Nevertheless, the amplitude at the resonant frequency changed slightly. Figure 15 and Table 5 show the S-parameters for the top and bottom positions of the PP tube loaded with two types of SUTs.

### 3.2. Semi-Solid Sample under Measurements

**Figure 16:**
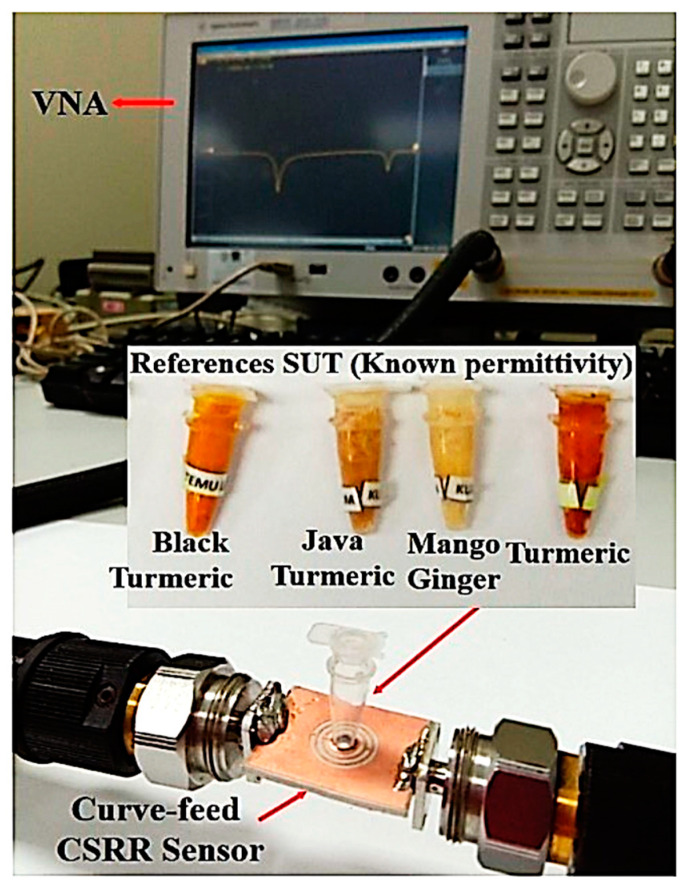
The SUTs experimental validation for the triple-rings CSRR sensor.

Various analyses of dielectric properties for reliability and validation of the sensor efficiency of semi-solid samples are measured, as shown in Figure 16. Four types of rhizomes from Zingiberaceace families, namely Java turmeric, Mango ginger, Black turmeric and Turmeric, were selected as they have the advantage of providing a particular scent relating to pharmacological material used as a drug. They are not just food or seasoning, but they are quite helpful products in traditional medicine as well. The availability of essential oil from the Zingiberaceace family for the medical, cosmetic and food industries strengthens the drive to validate the proposed sensor and determine its dielectric sensing ability.

**Figure 17:**
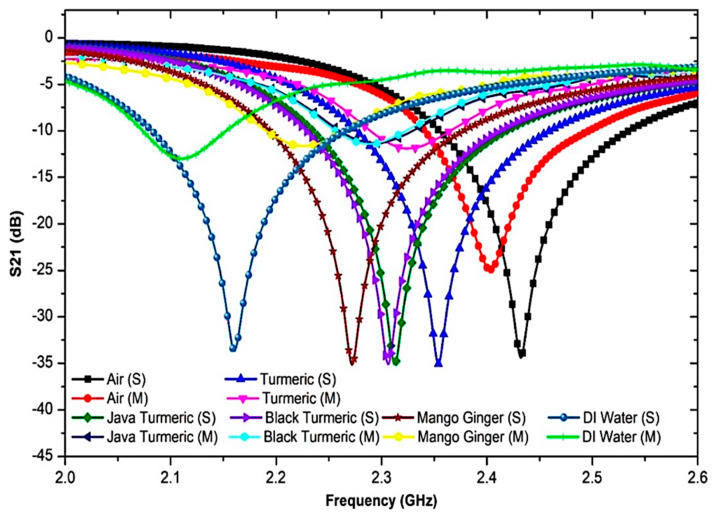
Comparison simulation and measurement of triple-rings CSRR sensor with several semi-solid samples.

Even before the unloaded sample, the retrieved resonance frequency for the sample becomes 2.5 GHz. When loaded, the resonance frequency is pushed down due to the higher value of the dielectric constant of the samples. Outcomes will be acquired and compared in Figure 17 and summarized in Table 6. As shown from the graph, the resonance frequency changes to a lower frequency as the dielectric sample has a higher permittivity value.

This indicates that there is a consistent pattern towards reduced simulation results in the maximum amplitude that contributes towards reducing the sensitivity of the sensor. The resonance frequency change is considered when information is connected with the permittivity of the SUTs. It shows that the resonance frequency is changed to a lower frequency by increasing the sample permittivity value. The changes in the frequency of resonance are based on the reaction of dielectric materials and the electric field distribution of the sensor in the perturbation technique. Table 6 displays simulated S-parameter data for the proposed sensor after loading a PP tube with a number of samples.

The polynomial fitting technique is employed for determining the unknown SUTs’ permittivity based on the reference permittivity (Aziz et al. [41]). The difference between the two reference datasets and the simulated permittivity was analyzed based on permittivity with the inclusion of SUTs. To calculate the permittivity of standard samples, the Frequency Change referring to each sample is used. The 2nd-order polynomial of the curve fitting technique is obtained in the equation below:

ε’ = −713.41*f*^2^ + 2957.8*f* − 2986.5 (3)

**Figure 18:**
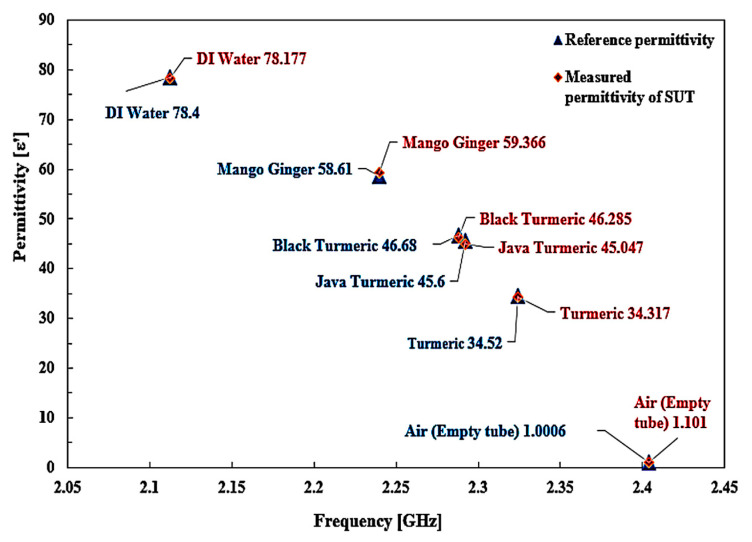
Comparison of the ideal and measured real part permittivity of several semi-solid SUTs.

The expression will be used to evaluate the real component of the material’s complex permittivity. From this stage, the particular equation will extract the unknown permittivity of any substance. The percentage error function and standard dielectric constant trend line of error are seen in Figure 18.

The permittivity values measured are tabulated in Table 7. Every single substance has specific values of permittivity. The frequency shift represents the properties of the substance itself. In other terms, the permittivity may be derived from the frequency-shifting response. Hence, the quality and safety of the materials may be calculated accurately, mostly on the basis of a useful permittivity parameter. The performance of the resonator is determined by measuring a dielectric sample’s permittivity in terms of the resonant shift, and the result shows great performance with different dielectric values. Each specific material has different permittivity values, and the frequency shift indicates the material properties themselves. It clearly shows that the resonance frequency has changed according to the increasing value of ε’ of the SUTs. Based on the result, with a minor change in the frequency shift, the Curve-feed CSRR sensor can detect and characterize materials.

Interestingly, compared to the analysis in reference value, the real permittivity of SUTs was very close to the same samples tested using the proposed triple-rings CSRR sensor. This proposed approach has a tolerance average of ±2.38% error detection of the Curve-feed CSRR sensor with minimum and maximum errors of 0.28% and 10.03%, respectively. The error detection is better than that of the commercial sensor by ±18.34%. However, the dimensions are difficult to measure accurately owing to several practical challenges in the production process, which have slightly different dimensional parameters compared to the simulation model. Further extensive changes to the responsiveness of the triple-rings CSRR sensor can be seen here in order to characterize materials for a planar structure.

**Figure 19:**
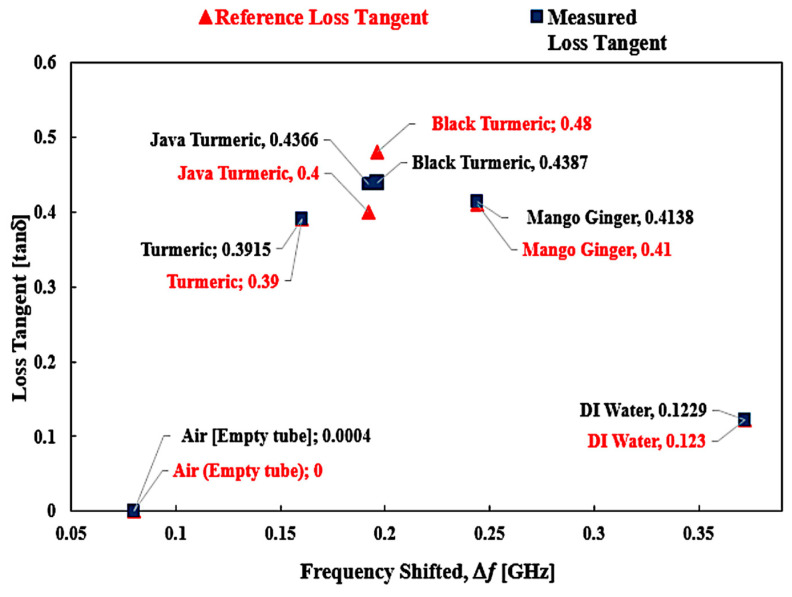
Comparison of the ideal and measured loss tangent of several SUTs (semi-solid).

A mathematical model of the curve fitting technique for the determination of loss tangent (tan δ), and imaginary portion (ε″) of the complex permittivity is used to monitor and analyze the frequency shift (Δ𝑓) of SUTs. A graphical description of the relationship between loss tangent percent error between the reference and measured values is highlighted in Figure 19.

Details for the SUTs’ reference loss tangent are shown by the marker of the red triangle shape point and measured as the blue square shape with the polynomial blue line polynomial fit of loss tangent. It can be found that the distribution of *tan δ* with the Δ*f* is not constant. Thus, the relationship between the two parameters may be described as the polynomial expression of the third order for producing an exact numerical model, as given by the equation below.

*tan δ* = 55.714(|Δ*f*|)^3^ − 55.213(|Δ*f*|)^2^ + 15.654(|Δ*f*|) − 0.9279 (4)

The outcomes of this analysis are summarized in Table 8. Based on the available data, it can be proposed that the triple-rings CSRR sensor provided a good minimum tolerance of measurement errors with the value ±4% compared to the commercial sensor with ±28.3%.

**Figure 20:**
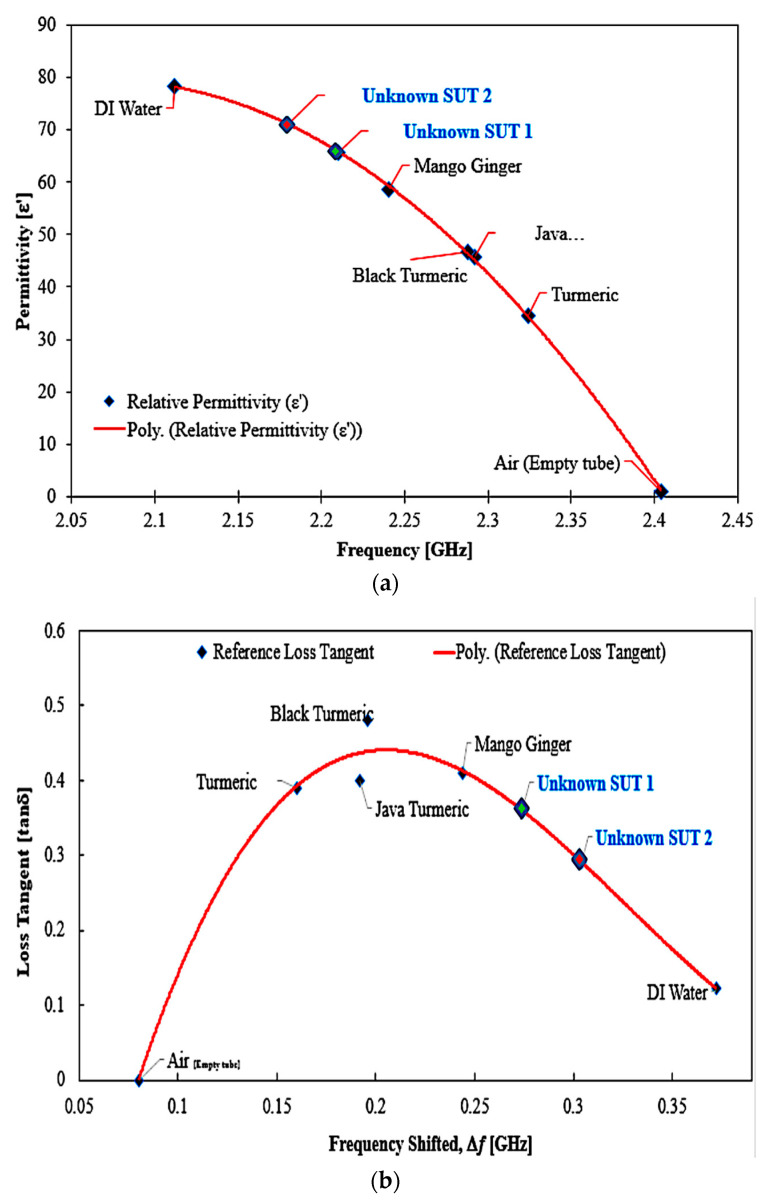
Unknown semi-solid SUTs: (**a**) real permittivity, (**b**) loss tangent versus resonant frequency extracted value, shown with known SUTs.

The reference as well as the proposed method demonstrated almost the same performance in loss tangent values. The java turmeric was 9.15% more inaccurate than other SUTs, 8.6% for black turmeric, 0.38% for turmeric and 0.93% for mango ginger. The air loss tangent assumes zero due to the standard loss tangent of the material and 0.08% value of water. For this study, due to the information that the PP tube has been utilized as a sample filled, air and water are taken into account, and the dielectric properties of the Zingiberaceace family obey the pattern of the dielectric properties of water. Every unknown semi-solid sample may be derived from the polynomial Equations (2) and (3) to calculate the value of real permittivity and loss tangent, respectively. This is dependent on the frequency values of the two unknown SUTs as shown in Figure 20.

**Figure 21:**
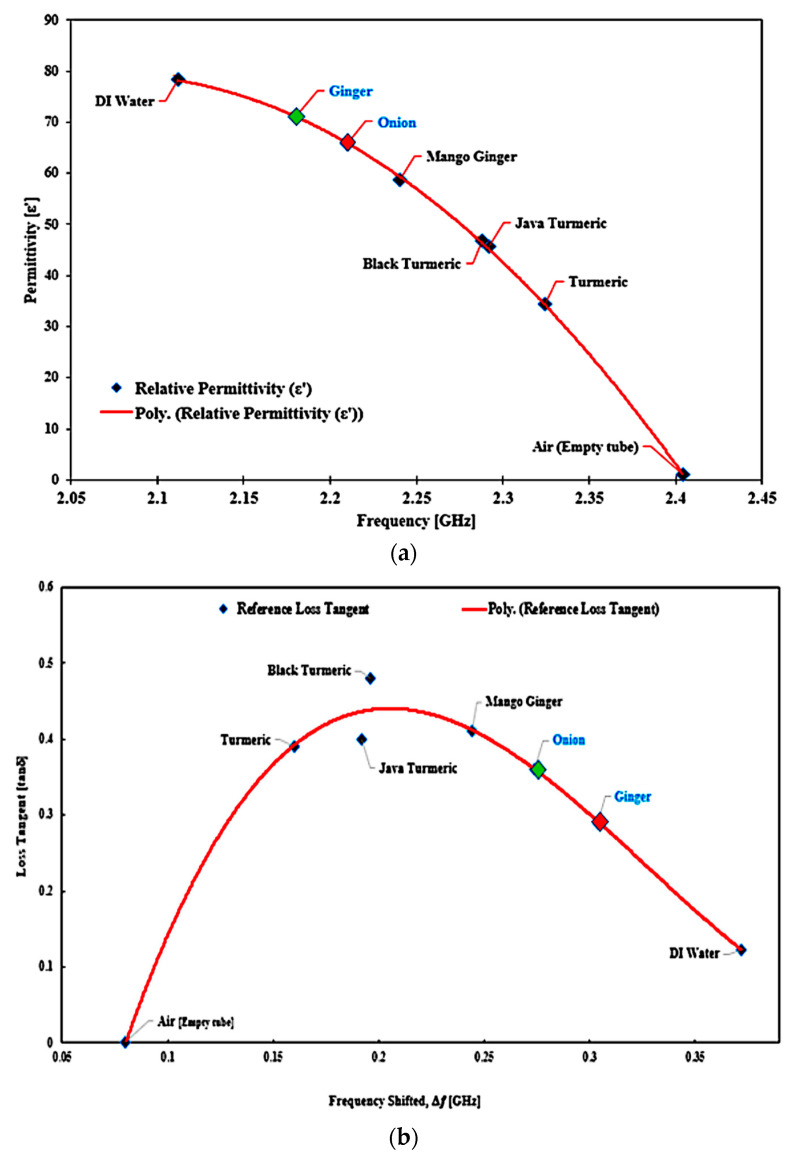
The position value of unknown SUTs (semi-solid) based on frequency and calculated (**a**) real permittivity and (**b**) loss tangent.

The calculation is obtained and the unknown sample is defined as having almost the same values reported by [42,43,44] as being onion and ginger, respectively. The experiment results for real permittivity and loss tangent determined by polynomial equations for each SUTs are compared in Table 9 and illustrated in Figure 21.

### 3.3. Sensitivity

The resonant frequency response is based on the material*’*s dielectric constant. The electrical field of the resonator will interface when the SUT is installed on the maximum electrical fields of the triple-rings CSRR sensor. It was found that the resonant frequency will change. The differential shift in the resonant frequency (Δ*f*) and the related permittivity (Δ𝜀) can be calculated using Equation (5) to determine the sensitivity value, and it can be calculated based on the equation [45]:

𝑆 = ∆𝑓/∆ε′(5)

where Δ*f* is the proportional difference between unloaded and loaded SUT, ∆𝑓 = (𝑓_o_ − 𝑓_s_)/𝑓_s_. Meanwhile, the variation of permittivity Δε is represented by where air and SUT’s 0𝑠𝑠 permittivity, ∆ε′ = (ε′ − (ε′)). The fractional changes in the resonating frequency have been measured for efficient permittivity, described as sensitivity (S), to assess the sensor*’*s performance. Owing to the relative changes in the changing rate of the sensor triple-rings CSRR, this contributes to the relative alteration of the permittivity of the samples, which is often used as a reference empty sample tube (SUT = Air). Table 10 shows the sensitivity of various solid SUTs.

The proposed sensor has greater sensitivity compared to #1 up to # 12 since it has larger e-fields. The presence of the triple-rings CSRR sensor*’*s electric field eventually influences the resonant frequency shift once the SUT permittivity is changed. The findings show that any improvements in the dielectric properties of the sample can impact the resonant frequency shifts and sensitivity of the sensor in the resonant perturbation technique. A comparison shows a competitive performance of the presented design in terms of compactness, Q-factor, and sensitivity as tabulated in Table 11.

## 4. Conclusions

This study examined a low-cost and highly efficient triple-rings microwave sensor working at 2.5 GHz for semi-solid material characterizations. The SUTs are filled into Polypropylene (PP) tube channels and loaded into the CSRR resonator center hole. The e-fields near the resonator will affect the interaction with the SUTs; a strong and harmonious electric field on resonance exists, and the measured transmission response varies significantly. The presented triple-rings CSRR sensor can specify a few standard semi-solid specimens and the concentrations of SUTs mixtures through detailed measurements. The RT/Duroid Roger 6002 has been chosen as the substrate due to low electricity loss and stable dielectric constant over frequency. A high-frequency structural simulator (HFSS) version 15.0 has been used to simulate the proposed design of a triple-rings CSRR. The suggested Curve-feed CSRR sensor offered the best performance with high accuracy and the lowest average error detection at 0.23%. The finalized triple-rings CSRR sensor has a miniaturized size and high sensitivity, which make it a good candidate for semi-solid material characterization.

## Figures and Tables

**Table 1:** Triple-rings design parameters.

Parameters	L	W	F_L_	F_W_	R_H_	R_W_	R_g_	R_s_	R_1_	R_2_	R_3_
Values (mm)	25	20	28	2.1	2	0.68	0.5	0.5	5.54	3.18	3.18

**Table 2:** The comparison of simulation performance of different rings of CSRR.

CSRR	Frequency (GHz)	Q-Factor	Insertion Loss, 𝑺_21_ (dB)	Electric Fields (v/m)
Single Ring	3.23	91	−23.476	9.8 × 10^3^
Double Ring	2.57	220	−24.949	1.33 × 10^4^
Triple Ring	2.5	520	−34.281	1.55 × 10^4^

**Table 3:** Simulation datasets of triple-rings CSRR design with several SUTs.

SUTs	Frequency (GHz)	S21 (dB)	Frequency Shifted (MHz)
Without tube	2.5	−34.2808	0
Air (empty tube)	2.432	−34.3829	68
Turmeric	2.354	−35.0751	146
Java Turmeric	2.313	−35.1014	187
Black Turmeric	2.306	−35.1009	194
Mango Ginger	2.272	−35.1594	228
DI Water	2.16	−33.6507	340

**Table 4:** Resonant frequency, Q—factor and S21 magnitude (dB) simulated and measured for triple-rings CSRR Sensor.

SUTs	Q-Factor	Simulation		Measurement	
		Frequency (GHz)	S_21_ (dB)	Frequency (GHz)	S_21_ (dB)
Unloaded	520	2.5	−34.281	2.484	−24.799
Loaded	230	2.432	−34.3829	2.404	−29.9078

**Table 5:** Comparative measurement results of semi-solid channel location (PP tube) for top and bottom triple-rings CSRR sensor.

SUTs	Top Sensor		Bottom Sensor	
	Frequency (GHz)	S_21_ (dB)	Frequency (GHz)	S_21_ (dB)
Air (Empty tube)	2.404	−24.9078	2.404	−23.0683
Turmeric	2.324	−11.9063	2.324	−13.8716
Java Turmeric	2.292	−11.3478	2.292	−11.9612
Black Turmeric	2.288	−11.4268	2.288	−13.7979
Mango Ginger	2.24	−11.6293	2.24	−13.5679
DI Water	2.112	−13.0072	2.112	−15.0731

**Table 6:** Comparison between the simulation and measurement of resonant frequency shift across the several SUTs.

SUTs	Relative Permittivity (*ε_r_*)	Simulation		Measurement	
		Frequency (GHz)	S_21_ (dB)	Frequency (GHz)	S_21_ (dB)
Air (Empty tube)	1.0006	2.423	−34.0156	2.404	−24.9078
Turmeric	34.52	2.354	−35.0751	2.324	−11.9063
Java Turmeric	45.6	2.313	−35.1014	2.292	−11.3478
Black Turmeric	46.68	2.306	−35.1009	2.288	−11.4268
Mango Ginger	58.61	2.272	−35.1594	2.24	−11.6293
DI Water	78.4	2.16	−33.6507	2.112	−13.0072

**Table 7:** Comparison of real permittivity and percentage error detection between the proposed and commercial sensors of several semi-solid SUTs.

SUTs	Frequency Shifting (GHz)	Reference Relative Permittivity	Proposed Sensor		* Commercial Sensor	
			Relative Permittivity (*ε’*)	Error (%)	Relative Permittivity (*ε^’^*)	Error (%)
Air (Empty tube)	2.404	1.0006	1.006	10.03	1.0093	0.969
Turmeric	2.324	34.52	34.317	0.59	52.43	51.88
Java Turmeric	2.292	45.6	45.047	1.21	54.60	19.74
Black Turmeric	2.288	46.68	46.285	0.85	48.74	4.41
Mango Ginger	2.24	58.61	59.366	1.29	41.28	29.57
DI Water	2.112	78.4	78.177	0.28	81.19	3.56
**Average Error**			**2.38%**		**18.34%**	

* Agilent 85070E dielectric probe.

**Table 8:** Comparison percentage error of loss tangent between proposed and commercial sensor of SUT (semi-solid).

SUTs	Frequency Shifting (Δ*f*)	Reference Ideal Loss Tangent	Proposed Sensor		* Commercial Sensor	
			Loss Tangent (*tan δ*)	Error (%)	Loss Tangent (*tan δ*)	Error (%)
Air (Empty tube)	0.08	0	0.0004	0	0.0001	0
Turmeric	0.16	0.39	0.3915	0.38	0.253	35.135
Java Turmeric	0.192	0.4	0.4366	9.15	0.252	36.905
Black Turmeric	0.196	0.48	0.4387	8.6	0.261	45.615
Mango Ginger	0.244	0.41	0.4138	0.93	0.383	6.702
DI Water	0.372	0.123	0.1229	0.08	0.197	45.43
**Average Error**			**4%**		**28.3%**	

* Agilent 85070E dielectric probe.

**Table 9:** The calculated complex permittivity of several known and unknown SUTs (semi-solid).

SUTs	*f* (GHz)	Δ*f* (GHz)	Reference			Calculated		
			*ε′*	*tan δ*	*ε″*	*ε′*	*tan δ*	*ε″*
Air (Empty tube)	2.404	0.08	1.0006	0	0	2.83	0.0004	0.0008
Turmeric	2.324	0.16	43.52	0.39	13.46	34.64	0.3915	12.5616
Java Turmeric	2.292	0.192	45.6	0.4	18.24	45.191	0.4366	19.7304
Black Turmeric	2.288	0.196	46.68	0.48	22.41	46.411	0.4387	20.3605
Mango Ginger	2.24	0.244	58.61	0.41	24.03	59.324	0.438	24.5483
Onion	2.21	0.274	64	0.218	14	65.781	0.3622	23.8259
Ginger	2.18	0.304	71.42	0.199	14.23	70.996	0.2936	20.8444
DI Water	2.112	0.372	78.4	0.123	9.64	78.222	0.1229	9.6135

**Table 10:** Sensitivity of the various SUTs.

SUTs	Frequency (GHz)	Δ*f* (MHz)	ε_r_	Δε_r_	S [MHz/εr]
Air (Empty tube)	2.404	80	1.0006	0	0
Turmeric	2.324	160	34.52	33.519	4.773
Java Turmeric	2.292	192	45.6	44.599	4.305
Black Turmeric	2.288	196	46.68	45.679	4.291
Mango Ginger	2.24	244	58.61	57.609	4.235
DI Water	2.112	372	78.4	77.399	4.806

**Table 11:** Detailed comparison of state-of-the-art technology of curve-feed CSRR sensor for material detections with the existing work of literature.

#	References	Sensors Sizes (mm)	Used Techniques	SUTs Samples	Frequency Band (GHz)	Q-Factor	Sensitivity (S)
1	[25]	80 × 40 × 0.8	Metamaterial coupling	Liquid	2.5	Not reported	0.27
2	[26]	80 × 25 × 0.8	Loss-compensated SRR	Glucose	1.156	190	Not reported
3	[27]	26 × 30 × 26.5	Waveguide with loop slot	Liquid	91	Not reported	Not reported
4	[28]	112.96 × 49.16 × 3.175	Multiple split-ring resonator	Liquid	2.1	525	Not reported
5	[33]	40 × 50 × 0.79	Two arms SRR	Solid	2.27	240	Not reported
6	[36]	38 × 35.4 × 15.73	GWCR	Liquid	5.96	66.8	0.156
7	[45]	25 × 30 × 1.54	CCSR	Liquid	2.4	Not reported	Not reported
8	[46]	30 × 25 × 1.6	CSSRRs	AIR, HDPE and PVC	5.35 and 7.99	267.5	0.04
9	[47]	46 × 46 × 1.6	OCSRRs	Liquid	0.9	Not reported	4.3
10	[48]	28 × 20 × 0.75	CSRR	Liquid	2.85 and 2.96	145	3.0
11	[49]	24 × 60 × 1.6	DS-SRR	Coal	4.75	Not reported	Not reported
12	[50]	70 × 70 × 1.6	Star-Slotted Patch	Oil	2.68	37.36	1.87
**This work**		**25 × 20 × 1.52**	**Triple-rings CSRR**	**Semi-solid**	**2.5**	**520**	**4.806**

## Data Availability

Not applicable.
